# Dr. Sale’s Case of Obliteration of a Part of the Intestinal Canal, with a Rare and Unusual Formation

**Published:** 1842-11-19

**Authors:** Richard A. Sale

**Affiliations:** Bedford County, (Va.)


MEDICAL EXAMINER.

NEW SERIES.

No. 47.] PHILADELPHIA, NOVEMBER 19, 1842. [Vol. I.

*Case of Obliteration of a part of the Intestinal Canal, with a rare and unusual formation.* By Richard A. Sale, M. D.

On the 13th day of October last I was called to see a coloured man who was labouring under symptoms of colic. He had suffered from previous attacks of this disease, at various intervals during the last eight or ten years. His sufferings were great, and, notwithstanding the most prompt and decided measures were had recourse to, by myself and two other medical gentlemen, the case terminated fatally. But before his death all the symptoms of ileus were present. The abdominal viscera were examined by Dr. Davis and myself, twelve hours after death. The small intestines were greatly distended, and much larger than natural. At the junction of the ileum with the cæcum there was an entire obliteration of the former, for the space of some inches, leaving not the slightest vestige of a canal. Above the obliterated portion the bowels were gangrenous for some distance. The most remarkable feature, however, was that of a malformation, or an adventitious production, resembling in looks and structure a portion of bowel. This raw production was given off from the cæcum, nearly opposite the appendix vermiformis, (which was natural) of the size of the colon, occupying the pelvic region, about ten inches in length, surrounded by mesentery, containing fœcal mater, and terminating spirally in a cul-de-sac, which rested on the lower anterior wall of the rectum. It should be stated that during the progress of the case the introduction of the finger into the rectum furnished evidence of the existence of a tumour, occupying the position in which this pouched termination of the new bowel was found after death. Adhesions, more or less perfect, the result of peritoneal inflammation, had taken place between it and the parts in contact with it; and the remark is true of the contents of the cavity generally. The colon was uncommonly small, and very much strictured in several places. I will not offer any conjectures, as to what gave rise to this formation. It is impossible to surmise whether it existed at his birth, or whether from some extraordinary effort of nature, it was an adventitious formation. The obliteration of the bowel had, I presume, been gradually taking place for years, and was completed after his last attack, as his death did not occur until the twelfth day from the attack.

*Bedford County, (Va.)* Nov. 10, 1842.

